# Recombinant Expression and Characterization of the Cytoplasmic Rice β-Glucosidase Os1BGlu4

**DOI:** 10.1371/journal.pone.0096712

**Published:** 2014-05-06

**Authors:** Chen Rouyi, Supaporn Baiya, Sang-Kyu Lee, Bancha Mahong, Jong-Seong Jeon, James R. Ketudat-Cairns, Mariena Ketudat-Cairns

**Affiliations:** 1 School of Biotechnology, Institute of Agricultural Technology, Suranaree University of Technology, Muang District, Nakhon Ratchasima, Thailand; 2 School of Biochemistry, Institute of Science, Suranaree University of Technology, Muang District, Nakhon Ratchasima, Thailand; 3 Guizhou Institute of Upland Food Crops, Guizhou Academy of Agricultural Sciences, Guiyang, Guizhou, China; 4 Graduate School of Biotechnology and Crop Biotech Institute, Kyung Hee University, Yongin, Gyeonggi, Korea; 5 Department of Plant Molecular Systems Biotechnology, Kyung Hee University, Yongin, Gyeonggi, Korea; Institute of Molecular Genetics IMG-CNR, Italy

## Abstract

The Os1BGlu4 β-glucosidase is the only glycoside hydrolase family 1 member in rice that is predicted to be localized in the cytoplasm. To characterize the biochemical function of rice Os1BGlu4, the *Os*1*bglu*4 cDNA was cloned and used to express a thioredoxin fusion protein in *Escherichia coli*. After removal of the tag, the purified recombinant Os1BGlu4 (rOs1BGlu4) exhibited an optimum pH of 6.5, which is consistent with Os1BGlu4's cytoplasmic localization. Fluorescence microscopy of maize protoplasts and tobacco leaf cells expressing green fluorescent protein-tagged Os1BGlu4 confirmed the cytoplasmic localization. Purified rOs1BGlu4 can hydrolyze *p*-nitrophenyl (*p*NP)-*β*-d-glucoside (*p*NPGlc) efficiently (*k*_cat_/*K*_m_  =  17.9 mM^−1^·s^−1^), and hydrolyzes *p*NP-*β*-d-fucopyranoside with about 50% the efficiency of the *p*NPGlc. Among natural substrates tested, rOs1BGlu4 efficiently hydrolyzed β-(1,3)-linked oligosaccharides of degree of polymerization (DP) 2–3, and β-(1,4)-linked oligosaccharide of DP 3–4, and hydrolysis of salicin, esculin and *p*-coumaryl alcohol was also detected. Analysis of the hydrolysis of *p*NP-*β*-cellobioside showed that the initial hydrolysis was between the two glucose molecules, and suggested rOs1BGlu4 transglucosylates this substrate. At 10 mM *p*NPGlc concentration, rOs1BGlu4 can transfer the glucosyl group of *p*NPGlc to ethanol and *p*NPGlc. This transglycosylation activity suggests the potential use of Os1BGlu4 for *p*NP-oligosaccharide and alkyl glycosides synthesis.

## Introduction

Beta-glucosidases (β-d-glucoside glucohydrolases, E.C. 3.2.1.21) are enzymes that hydrolyze glycosidic bonds to release nonreducing terminal glucosyl residues from glycosides and oligosaccharides, and are classified among the glycoside hydrolases (GH) [1]. GH have thus far been classified to 133 families based on amino acid sequence and structural similarity, and the families that have similar catalytic domain structures and conserved catalytic amino acids, are grouped into clans (http://www.cazy.org) [2]. Of the GH families, those containing β-glucosidases include GH1, GH3, GH5, GH9, GH30 and GH116. Most plant β-glucosidases fall into GH1.

In plants, β-glucosidases are involved in a variety of processes, such as release of glucose from oligosaccharides derived from cell-wall polysaccharides, defense against herbivores and fungi by release of toxic compounds from inactive glycosides, activation of lignin precursors, release of phytohormones from inactive glycosides, release of scent compounds from involatile glycosides, and activation of glucose-blocked intermediates in metabolism [1]. The substrate specificity, tissue and subcellular localization, and conditions under which they come into contact with their physiological substrates determine the roles of these enzymes.

**Figure 1:**
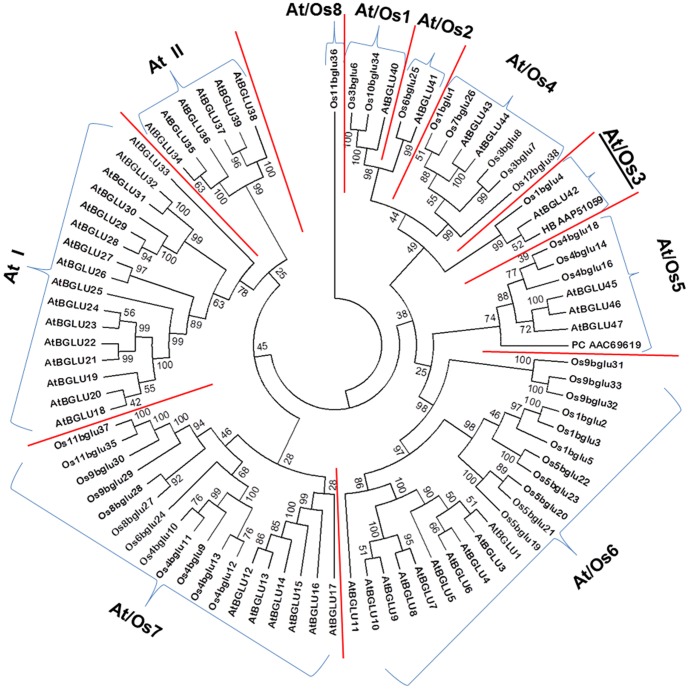
Protein sequence-based phylogenetic tree of rice and Arabidopsis glycoside hydrolase family GH1 enzymes. The phylogenetic clusters described by Opassiri et al. [3] are separated by red lines and labeled At/Os1-8, At I and At II. Os9BGlu36 was used to root the tree, so the Arabidopsis member of At/Os8, SFR2, is not shown. The Arabidopsis sequences are named as described by Xu et al. [37], while the rice members are named as described by Opassiri et al. [3]. The pine coniferin β-glucosidase (PC AAC69619) and rubber tree β-glucosidase (HV AAP51059), which are mentioned in the text, are shown labeled by the first letters of their genus and species and Genbank accession numbers. The tree was developed with the neighbor-joining method based on the protein sequence alignment made with the MUSCLE algorithm [38] implemented in MEGA 5.2 [39]. Cluster At/Os4 is emphasized with a larger underlined label, since it is the subject of the current work.

Forty rice genes homologous to GH1 β-glucosidase have been identified from rice genome databases, and 34 of these are likely to form active rice glycosidases [3]. The relationship between rice and *Arabidopsis thaliana* GH1 protein sequences have been described by a phylogenetic tree rooted by Os11BGlu36 (Figure 1), in which the GH1 members from rice and *Arabidopsis* were classified to 8 clusters containing members from both plants (At/Os1–8), and 2 clusters only found in *Arabidopsis* [3]. Since it is plausible that genes in the same cluster may have similar functions, we have set out to clarify the function of one representative gene to help understand the functions of the genes in each cluster.

So far, several rice β-glucosidases from different At/Os phylogenetic clusters have been isolated and characterized. Os3BGlu7 (rice BGlu 1) and its close relatives Os3BGlu8 and Os7BGlu26, from the At/Os4 phylogenetic cluster, were found to release glucose and mannose from oligosaccharides generated in cell wall remodeling at various stages of plant development [4], [5]. Another possible function of Os3BGlu7 is the release of the coenzyme pyridoxine from its glucose-conjugated storage form [4]. The At/Os1 cluster representative Os3BGlu6, in contrast to the At/Os4 isoenzymes described above, has little activity on β-1,4-linked oligosaccharides and prefers hydrophobic glycosides [6]. It was recently shown to have relatively high activity toward gibberellin GA4 1-O-acyl glucose ester compared to other rice enzymes [7]. At/Os7 cluster representative Os4BGlu12 was originally identified to act on oligosaccharides, such as those released in wounding, but was recently implicated in the release of the phytohormones tuberonic acid and salicylic acid [8], [9], similar to its close relative Os4BGlu13 (also called rice tuberonic acid β-glucosidase 1, OsTAGG1) [10], [11], suggesting these enzymes could have multiple roles in phytohormone and oligosaccharide metabolism. Another rice enzyme with a possible role in phytohormone metabolism is the At/Os6 representative Os9BGlu31 [12], which is a transglucosidase that can generate and breakdown the 1-O-acyl glucose esters of the phytohormones abscissic acid, auxins (indole acetic acid and naphthalene acetic acid), and gibberellin, in addition to those of other phenolic acids, such as ferulic acid.

Although no rice representatives of the At/Os2, At/Os5 and At/Os8 clusters have been characterized to date, the At/Os2 cluster is closely related to At/Os1, while the roles of At/Os5 and At/Os8 enzymes can be inferred from their *Arabidopsis* members [3], [13], [14]. The At/Os5 representatives *Arabidopsis* BGLU45 and BGLU46 were shown to hydrolyze monolignol glucosides [13], similar to a previously characterized pine tree coniferinβ-glucosidase [15], which also falls in this cluster (Figure 1). The *Arabidopsis* At/Os8 representative, sensitive to freezing 2 (SFR2), was shown to be the chloroplast galactolipid: galactolipid galactosyltransferase (GGGT) [14], implicating the sole rice At/Os8 representative, Os11BGlu36, in the same function. Thus, the current literature can be used to generate reasonable hypotheses for biochemical functions of members of the GH1 phylogenetic clusters At/Os1, 2, 4, 5, 6, 7, and 8.

Based on the above discussion, the only GH1 phylogenetic cluster shown in Figure 1 for which no functional inference may be made is At/Os3, which has one member each in rice and *Arabidopsis*. Os1BGlu4 is the only rice GH1 β-glucosidase without a predicted signal peptide, and is the sole rice representative in GH1 phylogenetic cluster At/Os3, which also includes *Arabidopsis* BGlu42 and *Hevea brasiliensis* latex cyanogenic β-glucosidase [3]. Therefore, we determined the substrate specificity and characteristics of Os1BGlu4, in order to explore the function of At/Os3 cluster members.

## Materials and Methods

### Bioinformatics analysis of *Os*1*bglu*4

The Os1BGlu4 cDNA sequence [accession number AK243365.1], from locus Os1g0897600 of the rice genome project, was retrieved from the National Center for Biotechnology Information website (http://www.ncbi.nlm.nih.gov/). Protein sequence analyses were done at the Expasy proteomics server (http://www.expasy.org/) [2].

### *E. coli* and plant expression vector construction

The coding sequence of the rice Os1BGlu4 gene was PCR-amplified with the forward primer (CACCATGGGGAGCACGGGGCGC), which introduced an *Nco*I site, and reverse primer (AGGGAATTCCTAGTTCATGTCAGC), which introduced an *Eco*RI site. Fourteen day-old rice leaf cDNA was used as template. The PCR product and pET32a(+) were digested with *Nco*I and *Eco*RI restriction endonucleases, purified with a QIA quick DNA gel extraction kit following the recommended protocol (QIAGEN, Hilden, Germany), and then ligated together by T4 DNA ligase. The recombinant pET32a(+)*Os1bglu4* was transformed into *E. coli* strain DH5α, selected on 50 µg/ml ampicillin. Plasmids were extracted and the insert sequenced completely by automated DNA sequencing (Macrogen, Seoul, Korea). The correct pET32a(+)*Os1bglu4* was transformed into Origami B(DE3) *E. coli*, and positive clones were selected on LB (Luria-Bertani) agar containing 15 µg/ml kanamycin, 12.5 µg/ml tetracycline and 50 µg/ml ampicillin.

### Recombinant protein expression and purification

To produce the protein, selected clones were grown at 37°C until the OD_600_ reached 0.5–0.6. Initial screening experiments showed that induction at 20°C gave the best production of active protein, and induction with 0.1 mM isopropyl β-d-thiogalactoside (IPTG) gave the highest production of active protein, but 0–0.5 mM IPTG induction gave similar levels. So, for protein production, the culture was incubated at 20°C for 16–18 h, with or without addition of 0.1 mM isopropyl β-d-thiogalactoside (IPTG). The induced cultures were centrifuged at 5,000 *g* for 10 min at 4 °C. The cell pellets were resuspended by vortexing in 5 ml protein extraction buffer (20 mM Tris-HCl buffer, pH 8.0, 200 µg/ml lysozyme, 1% TritonX-100, 40 µg/ml DNase I, 1 mM phenylmethylsulfonyl fluoride (PMSF)) per 1 g cell pellet, incubated at room temperature for 30 min, and disrupted by sonication. The soluble protein was recovered by centrifugation at 12,000 *g* at 4 °C for 10 min, and the activity of the soluble protein was examined.

The soluble protein containing thioredoxin-histidine-tag-recombinant Os1BGlu4 fusion protein (Trx-His_6_-rOs1BGlu4) was purified by immobilized metal affinity chromatography (IMAC) on TALON cobalt resin according to the manufacturer's instructions (Clontech, Palo Alto, CA, U.S.A.). The fractions with *p*NPGlc hydrolysis activity were pooled and concentrated with 10-kDa-cut-off Amicon regenerated cellulose ultra-centrifugal filters (Millipore, Billerica, MA, U.S.A.). The purified Trx-His_6_-rOs1BGlu4 fusion protein was digested with enterokinase according to the instructions of the manufacturer (New England Biolabs, Ipswich, MA, U.S.A.), and the mixture was applied to cobalt resin again and washed as described above, to purify the cleaved tag away from the free rOs1BGlu4. The flow-through and wash fractions containing activity were pooled and concentrated as described above. The purified rOs1BGlu4 was stored in 20 mM Tris-HCl, pH 8.0, and kept in −80 °C until use to characterize the biochemical properties. All protein samples were analyzed by 15% SDS polyacrylamide according to standard methods [4]. Protein concentrations were determined with the Bio-Rad (Hercules, CA, U.S.A.) protein assay kit with bovine serum albumin (BSA) as a standard.

### Optimum pH and pH stability

The pH optimum was determined by measuring the release of *para*-nitrophenol (*p*NP) from *p*NP-β-d-glucopyranoside (*p*NPGlc) in different 50 mM buffers with pH ranging from 4.0 to 11.0 [formate, pH 4.0; sodium acetate, pH 4.5–5.5; sodium phosphate, pH 6.0–7.5; Tris, pH 8.0–9.5; CAPS, pH 10.0–11.0] in 0.5 pH unit increments for 10 min [4]. The pH stability for the rOs1BGlu4 was determined by incubating 20 µg rOs1BGlu4 in 20 µl of buffers ranging from pH 4 to 10, as described above, at increments of 1.0 pH unit, for 10 min, 1, 3, 6, 12 and 24 h. The enzyme in each treatment was diluted 40-fold in 50 mM phosphate buffer, pH 6.5, and the activity was determined as described above.

### Optimum temperature and thermostability

The optimum temperature was determined by measuring the release of *p*NP from *p*NPGlc in 50 mM sodium phosphate, pH 6.5, at temperatures ranging from 5 to 90°C in 5°C increments for 10 min. The thermostability of the enzyme was checked by incubating the enzyme in 50 mM sodium phosphate, pH 6.5, at different temperatures ranging from 20 to 60°C at 10°C intervals for 10, 20, 30, 40, 50 and 60 min. At each time point, an aliquot of enzyme was removed and assayed at 30 °C as described above.

### Enzymatic analysis

The activity of the purified rOs1BGlu4 on *p*NP derivatives of monosaccharides was tested to determine the sugar specificity. In a 100* µ*l reaction volume, 0.25* µ*g rOs1BGlu4 was incubated with 1 mM substrates in 50 mM sodium phosphate, pH 6.5, at 30°C for 10 min. At the end of the reaction time, 70* µ*l of 0.4 M Na_2_CO_3_ was added to stop the reaction, and the absorbance at 405 nm was measured with a spectrophotometer [3].

The enzyme was tested with natural substrates and oligosaccharides, including cello-oligosaccharides (β-1,4-linked glucose) with degree of polymerization (DP) values of 2–6, laminari-oligosaccharides (β-1,3-linked) with DPs of 2–5, gentiobiose (β-1,6-linked glucobiose) and *p*NP-*β*-cellobioside. The reactions were stopped by boiling, and the glucose released was quantified by peroxidase/glucose oxidase (PGO) assay method (Sigma-Adrich, St. Louis, MO, U.S.A.) in 50 mM sodium acetate buffer, pH 5.0. For thin layer chromatography (TLC), a 50* µ*l reaction mixture, including 0.125* µ*g enzyme and 1 mM oligosaccharide in 50 mM sodium phosphate, pH 6.5, was incubated at 30°C for 20 min. The reaction mixtures (5* µ*l) were spotted onto silica-gel 60 F_254_ plates (Merck, Darmstadt, Germany) and chromatographed vertically with solvent consisting of ethyl acetate, acetic acid and water (2∶2∶1, v/v). The dried TLC plates were sprayed with 10% H_2_SO_4_ in ethanol (v/v) and baked at 120°C for 5 min to visualize the carbohydrate.

### Kinetic parameter determination

The Michaeilis-Menten constants (*K_m_*) and maximal velocities (*V_max_*), were calculated from the initial rates (*v*_0_) of hydrolysis of *p*NP-glycosides and oligosaccharides in triplicate assays. Substrate concentrations ranging from 1/4 to 4 fold of the apparent *K*_m_ value were reacted with enzyme concentrations and times that gave linear rates. The activity values for disaccharides were determined by dividing the amount of glucose released by two. The kinetic parameters were calculated by nonlinear regression of the Michaelis-Menten curves (*v*_0_  =  *V_max_* [S]/(*K_m_* + [S]) with the Grafit 5.0 program. The apparent *k*_cat_ values were calculated by dividing the *V*_max_ by the protein concentration.

### The inhibition study

The inhibitors were mixed to 10 mM with 1 mM *p*NPGlc in 50 mM sodium phosphate, pH 6.5, followed by adding 2.5 ng/µl rOs1BGlu4, and incubating for 10 min at 30°C. The reaction was stopped by adding 70* µ*l of 0.4 M Na_2_CO_3_, and the absorbance was read at 405 nm. The same reaction without the substrate was used as blank.

### Sequential hydrolysis of *p*NP-*β*-cellobioside

In order to investigate the sequential hydrolysis of *p*NP-*β*-cellobioside, 0.125 µg rOs1BGlu4 was incubated with 5 mM *p*NP-*β*-cellobioside in 50 µl 50 mM sodium phosphate, pH 6.5, at 30°C. The hydrolysis times ranged from 5 min to 1 h at 5 or 10 min intervals. The reaction mixture was sampled at the designated time points and the reaction was stopped by boiling. A 2 µl aliquot of each reaction mixture was spotted on a silica gel-coated F_254_ aluminum TLC plate (Merck) and chromatographed vertically with a solvent that consisted of ethyl acetate, methanol and water (7∶2.5∶1, v/v). The plate was first viewed under UV light to detect the *p*-nitrophenol moiety, followed by spraying with 10% H_2_SO_4_ in ethanol (v/v) and baking at 120°C for 5 min to visualize the sugar.

### Transglycosylation activity of rOs1BGlu4

In order to investigate the transglycosylation activity of the rOs1BGlu4, *p*NPGlc was used as the glucosyl group donor, while ethanol and *p*NPGlc were used as glucosyl group acceptors. Reactions contained 10 mM *p*NPGlc as donor, 0.125 mg rOs1BGlu4 and different concentrations of ethanol acceptor (10%, 20%, 30% and 50%), in 50 mM sodium phosphate, pH 6.5, and ran for 1 h, then were stopped by boiling 5 min. The reaction mixtures were spotted onto the TLC plate, which was developed with a solvent of 7∶3∶0.5 chloroform: methanol: water, then visualized by fluorescence and sulfuric acid charring, as described above.

The effect of different incubation times and *p*NPGlc concentrations on production of the transglycosylation products were evaluated. Reaction mixtures containing 0.5, 5, 10, 20 and 40 mM *p*NPGlc concentrations were reacted for 1, 2 and 3 h incubation times. The reaction mixtures were loaded onto the TLC plate and developed as described above.

### Subcellular localization

To examine the subcellular localization of Os1BGlu4, the entire open reading frames with or without the stop codon were amplified by PCR using a proofreading DNA polymerase (SolGent, Daejeon, Korea). The primer pairs used for PCR amplification were: 5′-CACCATGGGGAGCACGGGGCGCGACG-3′ as forward primer and 5′- CTAGTTCATGTCAGCTTTGTTC-3′ for N-terminal GFP fusion or 5′- GTTCATGTCAGCTTTGTTCTC-3′ for C-terminal fusion as reverse primer, respectively. The respective PCR products were cloned into the pENTR/D-TOPO vector (Invitrogen, Carlsbad, CA, U.S.A.) and recombined into the p2FGW7 vector for N-terminal GFP fusion and the p2GWF7 vector for C-terminal GFP fusion with LR clonase (Invitrogen) [17]. The resulting constructs, *GFP-Os1BGlu4* and *Os1BGlu4-GFP*, were introduced into maize mesophyll protoplasts by polyethylene glycol-mediated transformation [16].

The respective PCR products in the pENTR/D-TOPO vector were also cloned into the pH7WGF2 vector for N-terminal GFP fusion and the pH7FWG2 vector for C-terminal GFP fusion by LR clonase recombination (Invitrogen) [17]. The resulting fusion constructs were introduced into a tobacco leaf with P19 by an *Agrobacterium*-mediated infiltration method [18].

Expression of the fusion constructs was monitored with a confocal microscope (LSM 510 META, Carl Zeiss, Oberkochin, Germany) at various times after transformation. Chlorophyll autofluorescence and propidium iodide staining were used as markers of chloroplasts and nuclei, respectively. Total proteins extracted from transformed tobacco leaves transfected with the *GFP* fusion constructs were electrophoresed on a 10% SDS-PAGE gel and immunoblotted with an anti-GFP antibody (sc-8334, Santa Cruz Biotechnology, Santa Cruz, CA, U.S.A.).

### Transcription analysis in wounded rice leaves

To induce wounding stress, 10-day-old rice (*Oryza sativa* L. cv. Yukihikari) seedling leaves were gently crushed from the top to the bottom at 1 cm intervals with a blunt plastic ruler. Total RNA was extracted from stressed rice leaves after 10, 30, 60 and 180 min, according to the instructions of the TaKaRa MiniBEST Plant RNA Extraction Kit. The RNA was reverse transcribed to cDNA with PrimeScript RT reverse transcriptase and oligo-d(T) primer (Takara Bio Inc., Shiga, Japan).

The *Os1bglu4* qRT-PCR primers, RT-f (GTGGAGAGAATAGAAAAATGG), which spans exons 9 and 10, and RT-r (CTCATCCATGCCATTCTCAG), which spans exons 11 and 12, were designed to avoid amplification of contaminating genomic DNA in the cDNA template. The actin primers (Actin-f: TGC TATGTACGTCGCCATCCAG and Actin-r: AATGAGTAACCACGCTCCGTCA) were used to detect the *actin* gene cDNA [19]. The qRT-PCR reaction was prepared with SYBR *Premix Ex Taq* II (Takara). A Bio-Rad CFX96 real-time PCR detection system was used to amplify the cDNA and detect the product. The PCR reaction was initiated with denaturation at 95°C for 30 s, followed by 39 cycles of denaturation at 95°C for 5 s, annealing and extension at 60°C for 30 s. The fluorescence signal was read at the end of each extension step. Subsequently, a melting curve was generated to verify the specificity of the PCR products. The relative expression levels were calculated from the C_T_ values by the 2^−ΔΔC^_T_ method [20].

## Results and Discussion

### Bioinformatic analysis of rice *Os*1*bglu*4

The *Os1bglu4* coding sequence includes a 1449 bp open reading frame, which encodes a 483 amino acid long protein. The Os1BGlu4 protein sequence has the conserved catalytic core and active site residues of glycoside hydrolase family GH1, suggesting it should be an active β-glucosidase. The deduced protein was predicted by Signal P [21] to have no signal peptide and the subcellular localization predicted by PSORT is in the cytoplasm [4]. Unlike most putative rice β-glucosidases, which contain predicted secretary pathway signal sequences, Os1BGlu4 should exist in the cytoplasm [3]. The pI and molecular weight are predicted to be 5.16 and 55.3 kDa, respectively. Consistent with its cytoplasmic localization, no N-glycosylation site was predicted by NetNGlyc [22].

Analysis of the dbEST expressed sequence tag database (UniGene Os.18285) indicates that the *Os*1*bglu*4 gene is expressed in callus [18 transcripts per million (TPM)], flower (7 TPM), leaf (22 TPM), stem (16 TPM), and has the strongest expression in root (72 TPM) and panicle (51 TPM).

### Expression, extraction and purification of the Trx-His_6_-rOs1BGlu4

It has been reported that the substrate specificities and kinetic parameters of recombinant HvBII and HvBII isolated from germinated seeds were very similar [23], [24], suggesting that the substrate specificities determined for the enzyme expressed in the *E. coli* expression system are likely to approximate those of the corresponding native enzymes *in vivo*. In fact, when Os4BGlu12 expressed in *E. coli* and *Pichia pastoris* were compared with native protein from rice panicles, small differences in relative rates were seen for proteins from each preparation, but they all hydrolyzed the same substrates [9]. Therefore, the plant enzyme hydrolysis activity parameters were assessed through the recombinant enzyme expressed by *E. coli*.

**Figure 2:**
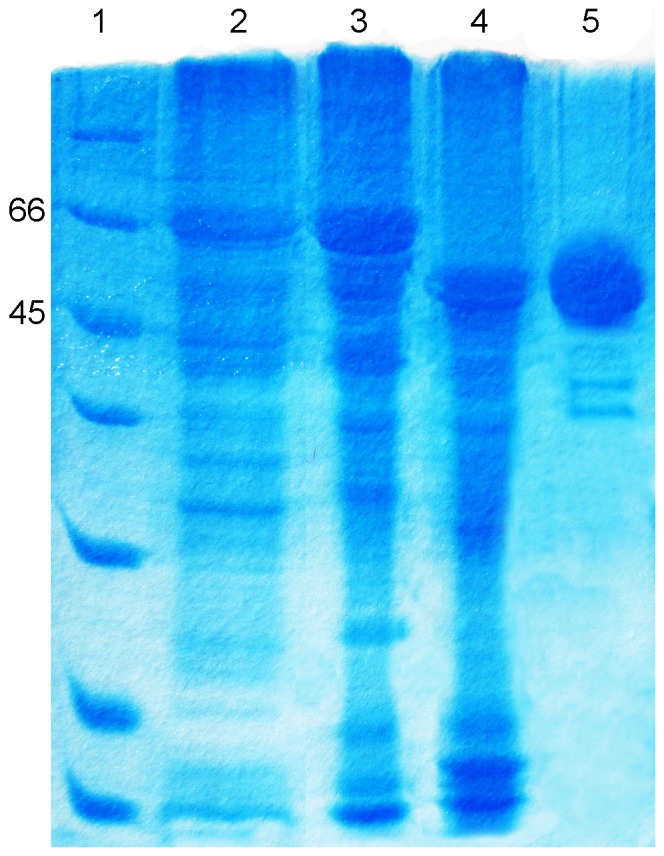
SDS-PAGE profiles of recombinant Os1BGlu4 expressed in Origami B(DE3). Lane 1, standard protein marker (Bio-RAD); Lane 2, crude Trx-His_6_-rOs1BGlu4; lane 3, purified Trx-His_6_-rOs1BGlu4; lane 4, Trx-His_6_-rOs1BGlu4 cleaved by enterokinase; lane 5, purified rOs1BGlu4. The numbers 45 and 66 indicate the molecular weights in kDa of the most relevant protein standards. Each lane was loaded with 8 µl of the sample mixed with 2 µl 5× sample dye.

When the recombinant Trx-His_6_-rOs1BGlu4 was expressed in *E. coli* Origami B(DE3), an intense band was observed at 66 kDa on SDS-PAGE (Figure 2, lane 2), and approximately 30% pure Trx-His_6_-rOs1BGlu4 was obtained upon purification by the initial IMAC step (Figure 2, lane 3). Approximately 2.8 mg of purified Trx-His_6_-rOs1BGlu4 was obtained per liter of bacterial expression culture. The purified Trx-His_6_-rOs1BGlu4 was cut by enterokinase and the recombinant Os1BGlu4 (rOs1BGlu4), which is about 55 kDa, and the thioredoxin tag were released (Figure 2, lane 4). The observed size of rOs1BGlu4 matches the predicted molecular weight (55.3 kDa). After a second IMAC purification step, approximately 85% pure rOs1BGlu4 was obtained, as estimated by photodensitometry of the SDS-PAGE gel (Figure 2, lane 5).

### pH optimum and pH stability of rOs1BGlu4

**Figure 3:**
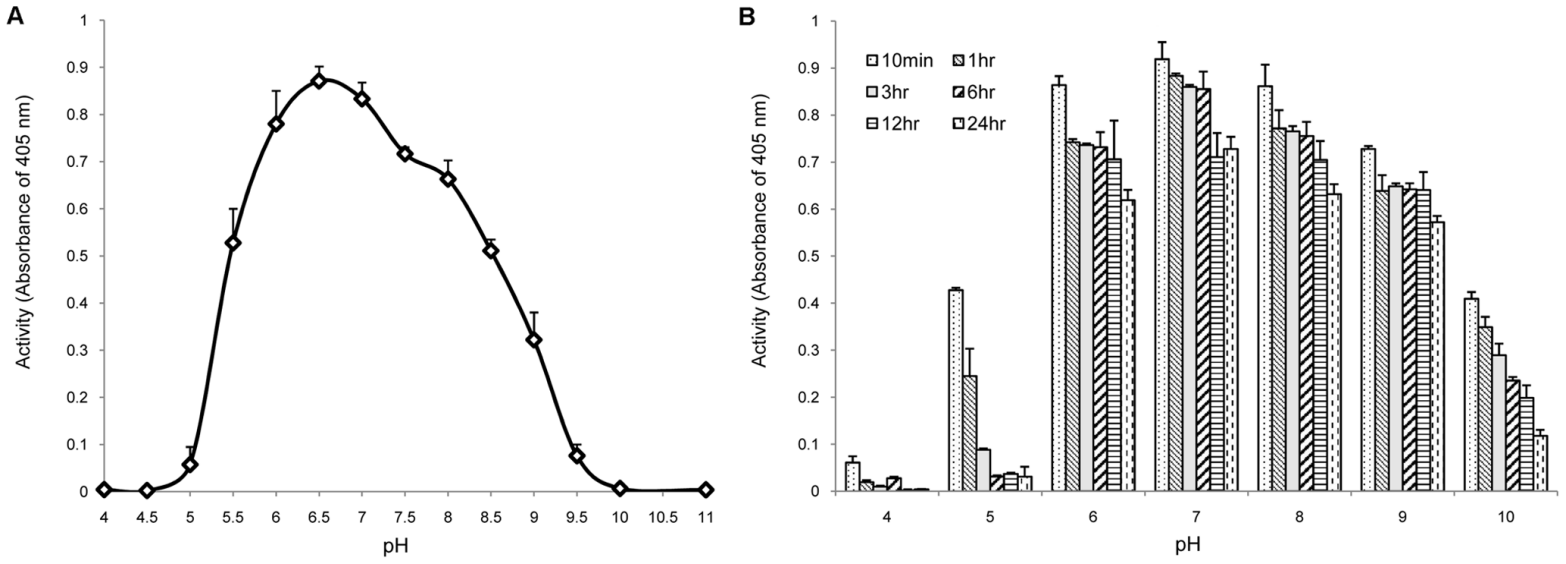
The pH optimum and pH stability of rOs1BGlu4 hydrolysis activity. A. pH optimum determination: rOs1BGlu4 (0.25 µg) was assayed with 1 mM *p*NPGlc in different 50 mM pH buffers (formate, pH 4.0; sodium acetate, pH 4.5–5.5; sodium phosphate, pH 6.0–7.5; Tris, pH 8.0–9.5; CAPS, pH 10.0–11.0) at 30°C for 10 min. B. pH stability evaluation: rOs1BGlu4 (20 µg) was incubated in the buffers described above for 10 min, 1, 3, 6, 12 and 24 h, then diluted 40-fold in 50 mM phosphate buffer, pH 6.5, and the activity was determined. The data are provided as mean + SE.

The hydrolysis activity of rOs1BGlu4 was highest at pH 6 to 7 (Figure 3A), dropped around 50% at pH 5.5 and 7.5, and was negligible from pH 5.0 downward. This pH optimum is higher than many other plant β-glucosidases (Os3BGlu6, pH 5.0; Os3BGlu8, pH 5.0; Os7BGlu26, pH 4.5; Os3BGlu7, pH 5.0; rHvBII, pH 4.0) [4], [6], [24], [25], and likely reflects the near neutral pH environment of the cytoplasm, where Os1BGlu4 is predicted to localize [26]. In contrast, the rice β-glucosidases listed above are expected to be in the apoplast or another acidic compartment. The rOs1BGlu4 had relatively high activity at pH 6.0, 7.0 and 8.0 after incubation with different pH buffers for up to 24 h (Figure 3B).

### Temperature optimum and thermostability of rOs1BGlu4

**Figure 4:**
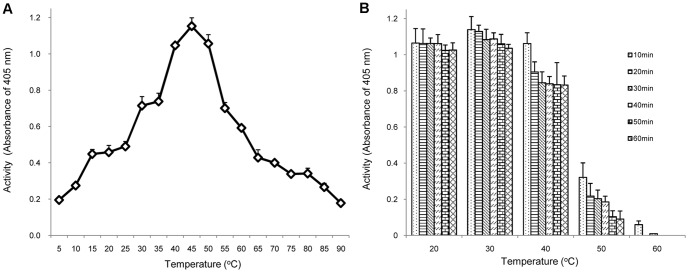
The temperature optimum and thermostability of rOs1BGlu4. A. Temperature optimum: rOs1BGlu4 (0.25 µg) was assayed with 1 mM *p*NPGlc in phosphate buffer, pH 6.5, at the designated temperature for 10 min. B. Evaluation of thermostability: the enzyme was incubated in phosphate buffer, pH 6.5, at temperatures ranging from 20°C to 60°C for 10, 20, 30, 40, 50 and 60 min. At each time point, an aliquot was removed and assayed at 30°C. The data are provided as mean + SE.

As the temperature increased from 5°C to 45°C, the activity of rOs1BGlu4 increased correspondingly in a 10 min reaction. However, as the temperature continued to increase from 45°C to 90°C, the activity of the rOs1BGlu4 decreased (Figure 4A). The optimum temperature was 45°C, which corresponded to the results of many plant β-glucosidases that have the temperature optima near 50°C [3], [27]. However, some β-glucosidases have higher optimal temperatures, such as the Thai rosewood and *Dalbergia nigrescens* β-glucosidases, which have a temperature optima of 60 and 65°C, respectively [28], [29]. Figure 4B shows that the rOs1BGlu4 was stable at 20 °C and 30 °C for up to 1 h incubation. The rOs1BGlu4 lost about 20% of its activity when incubated at 40 °C for 20 min, and was unstable at 50 °C and 60 °C.

### Substrate specificity and kinetic analysis

**Figure 5:**
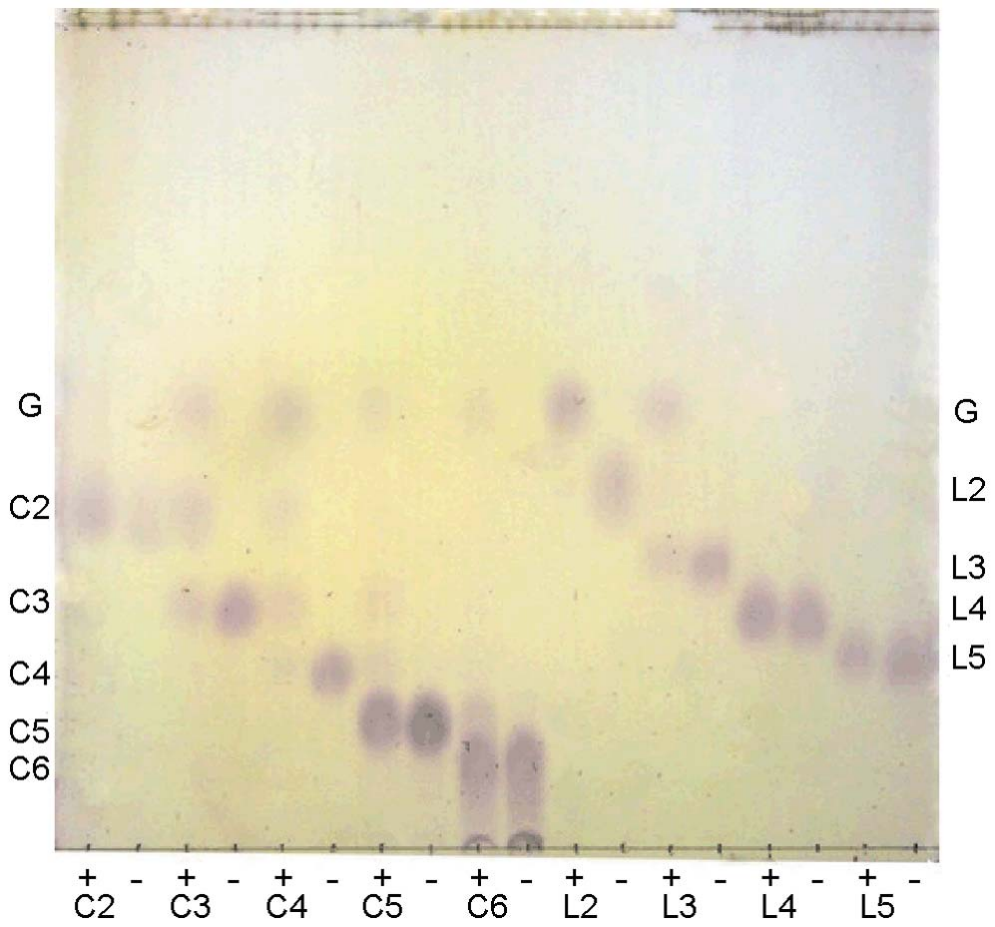
TLC of hydrolysis products of rOs1BGlu4 with cello-oligosaccharides and laminari-oligosaccharides. In each 50 µl reaction, 0.125 µg rOs1BGlu4 was incubated with 1 mM oligosaccharide in 50 mM sodium phosphate, pH 6.5, at 30 °C for 20 min. Samples were incubated with (+) and without (−) enzyme. Then, 2 µl of the reaction was spotted onto the TLC plate. Standards and substrates are: G, glucose; C2, cellobiose; C3, cellotriose; C4, cellotetraose; C5, cellopentaose; C6, cellohexaose; L2, laminaribiose; L3, laminaritriose; L4, laminaritetraose and L5, laminaripentaose.

The activities of the purified rOs1BGlu4 toward *p*-nitrophenyl glycosides, oligosaccharides and natural substrates are summarized in Tables 1 and 2. Figure 5 shows its cleavage of oligosaccharides. The rOs1BGlu4 hydrolyzed *p*NP glycosides, including *β*-d-glucopyranoside, *β*-d-fucopyranoside, *β*-d-galactopyranoside, *β*-cellobioside, *α*-l-arabinopyranoside, *β*-d-mannopyranoside and *β*-d-xylopyranoside, but hydrolysis of *p*NP-*α*-d-glucopyranoside, *p*NP-N-acetyl-*β*-d-glucosaminopyranoside, *p*NP-*β*-l-fucopyranoside, and *p*NP-*α*-l-fucopyranoside was not detectable. Among the monosaccharide *p*NP-glycosides, rOs1BGlu4 hydrolyzed *p*NPGlc most efficiently with catalytic efficiency (*k*_cat_/*K*_m_) of 17.9 mM^−1^·s^−1^, and *p*NP-*β*-d-fucopyranoside was hydrolyzed at 52.0% of the rate of *p*NPGlc, although the two substrates had the same *K*_m_ value. In addition, rOs1BGlu4 hydrolyzed *p*NP-*α*-l-arabinopyranoside, *p*NP-*β*-d-mannopyranoside, and *p*NP-*β*-d-galactopyranoside with *k*_cat_/*K*_m_ values that were 7.6%, 3.1%, and 2.4% that of *p*NPGlc, respectively. These results showed that rOs1BGlu4 is not stringent for glucose in the -1 subsite, where the non-reducing glycosyl moiety is bound. This property is similar to many GH1 and GH5 β-glucosidases, such as the rice Os3BGlu7 [4], Os4BGlu12 [3], and GH5BG enzymes [30].

**Table 1:** Kinetic parameters of rOs1BGlu4 in the hydrolysis of *p*NP glycosides and oligosaccharides.

Substrate	*k_cat_* (s^−1^)	*K_m_* (mM)	*k_cat_*/*K_m_* (mM^−1^·s^−1^)
*p*NP-glycosides			
*p*NP-*β*-D-glucoside	12.76±0.18	0.71±0.02	17.9
*p*NP-*β*-D-fucoside	6.61±0.08	0.71±0.02	9.34
*p*NP-α-L-arabinoside	0.52±0.00	0.38±0.02	1.37
*p*NP-*β*-D-galactoside	3.16±0.06	7.33±0.32	0.43
*p*NP-*β*-D-mannoside	1.25±0.02	2.24±0.03	0.56
oligosaccharides			
*p*NP-*β*-cellobioside	3.44±0.18	0.14±0.01	24.36
Laminaribiose	4.67±0.09	0.38±0.02	12.45
Laminaritriose	3.37±0.55	0.60±0.03	5.63
Cellobiose	0.58±0.01	19.0±0.5	0.03
Cellotriose	2.74±0.06	0.59±0.03	4.64
Cellotetraose	2.27±0.03	0.26±0.01	8.73
Cellopentaose	2.15±0.03	1.07±0.04	2.01
Cellohexaose	1.08±0.02	1.10±0.05	0.99

**Table 2:** Natural substrates hydrolyzed by rOs1BGlu4.

Substrate	glucose	Substrate	glucose
O-glycosides			
Salicin	+	Methyl-*β*-D-glucoside	-
Esculin	+	n-hepty-*β*-D-glucoside	-
*p*-Coumaryl alcohol glucoside	+	n-octyl-*β*-D-glucoside	-
Linamarin	-	epigenin-7-glucoside	-
d-Amygdalin	-	Indoxyl-*β*-D-glucoside	-
Trans-zeatin glucoside	-	Genistin	-
Daidzin	-	Quercetin-3-glucoside	-
Arbutin	-	GA_4_ glucose ester	-
Coniferin	-	Naringin	-
C-, N- S- glycosides			
Mangiferin	-	Uridine	-
Sinigrin	-		
Oligosaccharides			
α-Lactose	-	Lactulose	-
Palatinose	-	Maltose	-
Gentiobiose	-		

“+” glucose was observed on TLC; “-” glucose was not observed on TLC.

**Figure 6:**
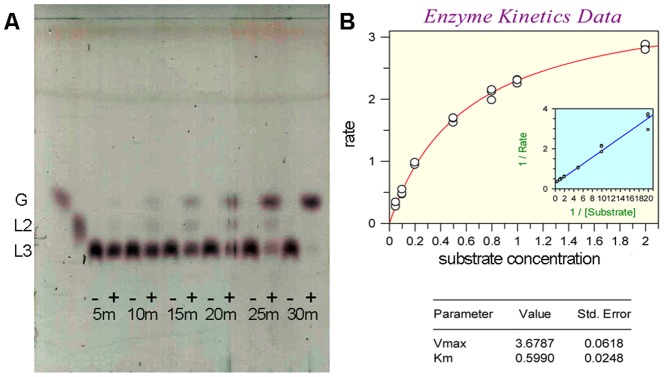
Evaluation of laminaritriose hydrolysis. A. Thin layer chromatographic evaluation of products of laminaritriose hydrolysis at different time points. rOs1BGlu4 (0.125 µg) was incubated with 1 mM laminaritriose in 50 mM sodium phosphate, pH 6.5, at 30 °C from 5 to 30 min (5 m–30 m). Samples were incubated with (+) and without (-) enzyme, then evaluated by silica gel TLC with sulfuric acid staining. The positions of glucose (G); laminaribiose (L2); and laminaritriose (L3) are marked. B: Kinetic data for laminaritriose hydrolysis. The Michaelis–Menten curve and inset Lineweaver-Burk plot are shown, along with the derived kinetic parameters and standard errors.

The rOs1BGlu4 β-glucosidase hydrolyzed β-(1,3)-linked oligosaccharides with DP of 2-3 and β-1,4-linked oligosaccharides with DP values of 2-6 at different rates, but not the β-(1,6)-linked disaccharide gentiobiose (Tables 1 and 2). Laminaribiose was most efficiently hydrolyzed (*k*_cat_/*K*_m_ of 12.5 mM^−1^·s^−1^), while laminaritriose (Figure 6) was relatively poorly hydrolyzed (*k*_cat_/*K*_m_ of 5.63 mM^−1^·s^−1^) and longer β-(1,3)-linked oligosaccharides were not hydrolyzed. In contrast, among β-1,4-linked oligosaccharides, cellobiose was the most poorly hydrolyzed and cellotetraose most efficiently hydrolyzed, with a catalytic efficiency (*k*_cat_/*K*_m_) of 8.7 mM^−1^·s^−1^, followed by cellotriose, cellopentaose, cellohexaose and cellobiose. In comparison, *p*NP-*β*-cellobioside was the most efficiently hydrolyzed substrate tested, with a *k*_cat_/*K*_m_ value of 24.4 mM^−1^·s^−1^. The decreasing efficiency of hydrolysis of cellopentaose and cellohexaose suggests that the rOs1BGlu4 has four subsites for binding of *β*-1,4-linked glucosyl residues. In TLC, only hydrolytic products were observed in rOs1BGlu4 reactions with 1 mM laminari-oligosaccharides and cello-oligosaccharides, and no transglycosylation products were detectable (Figure 4).

Cello-oligosaccharides and laminari-oligosaccharides were reported to be hydrolyzed by *β*-glucosidases that may be involved in cell-wall related processes. For example, rice Os3BGlu7 (BGlu1), Os3BGlu8, and Os7BGlu26 and Os4BGlu12 [5], [11]. Since Os1BGlu4 was predicted to be localized in the cytoplasm, it is surprising that it can hydrolyze these oligosaccharides.

Among other possible glycoside and oligosaccharide substrates tested (Table 2), only salicin, esculin and *para*-coumaryl alcohol glucoside (*p*CAG) were hydrolyzed by rOs1BGlu4, as observed from TLC. Due to their antioxidant properties, hydrolysis of these compounds could not be detected by the PGO assay. The rOs1BGlu4 was clustered with a *Hevea brasiliensis* latex cyanogenic β-glucosidase [3], but the plant cyanogenic glucosides linamarin and amygdalin were not hydrolyzed by rOs1BGlu4.

### Inhibition study

Table 3 indicates that HgCl_2_, δ-glucono-lactone, FeCl_3_, and CuSO_4_ and 1% SDS had strong inhibitory effects on the activity of rOs1BGlu4. At 10 mM concentration, HgCl_2_, δ-glucono-lactone and FeCl_3_ were able to inhibit almost 100% of the hydrolysis activity of rOs1BGlu4. In contrast, EDTA, CoSO_4_ and MnSO_4_ had no inhibitory effects on the hydrolysis activity of rOs1BGlu4 at 10 mM. When the concentration of HgCl_2_ was decreased to 0.05 mM, the inhibitory effect was still 100%, while 18% of the activity was recovered when the HgCl_2_ was diluted to 0.01 mM. For the δ-glucono-lactone, 62% of the activity was recovered when it was diluted to 0.1 mM.

**Table 3:** Inhibition of rOs1BGlu4.

Inhibitor	Activity	Relative activity remaining (%)
Control rxn	8.1	100
EDTA	8.35	103
CoSO_4_	8.16	101
MnSO_4_	8.11	100
KCl	7.88	97
CaCl_2_	7.8	96
D-Galactose	7.55	93
D-Glucose	7.29	90
MgCl_2_	7.16	88
CdCl_2_	6.98	86
D-Xylose	6.9	85
D-Glucosamine	6.82	84
NiSO_4_	6.4	79
ZnCl_2_	6.36	79
Imidazole	5.92	73
Urea	5.91	73
D-Mannose	5.91	73
LiCl	5.88	73
L-Arabinose	4.96	61
PbCl_2_	4.16	51
CuSO_4_	2.49	31
1% SDS	0.43	5
FeCl_3_	0.12	2
10 mMδ-glucono-lactone	0.02	0
1.0 mM δ-glucono-lactone	1.13	14
0.1 mM δ-glucono-lactone	5.02	62
10 mM HgCl_2_	0	0
1.0 mM HgCl_2_	0	0
0.1 mM HgCl_2_	0	0
0.05 mM HgCl_2_	0	0
0.01 mM HgCl_2_	1.46	18

Concentration of inhibitors is 10 mM, unless otherwise stated.

### Sequential hydrolysis of *p*NP-*β*-cellobioside

**Figure 7:**
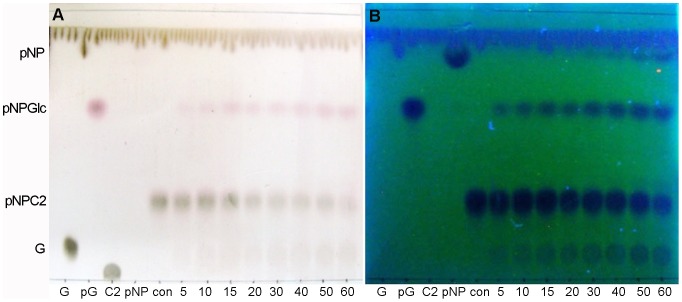
Sequential cleavage and transglycosylation of *p*NP -***β***-**cellobioside.** In a 50 µl reaction, 0.125 µg rOs1BGlu4 was incubated with 5 mM *p*NP-*β*-cellobioside in 50 mM sodium phosphate, pH 6.5, at 30°C. The reaction was sampled at the designated time points (5–60 min). Two microliters of the reaction were loaded onto the silica gel 60 F_254_ TLC plate. G, glucose; pG, *p*NPGlc; con, *p*NP-*β*-ellobioside in buffer without enzyme; C2, cellobiose; *p*NP, *p*-nitrophenol and 5–60, 5 minute to 60 minute reactions. A: TLC plate visualized by UV light. B: TLC plate visualized by carbohydrate staining.

Although rOs1BGlu4 could release *p*NP from *p*NP-*β*-cellobioside, it was hypothesized that this should result from two sequential reactions, the first releasing one glucose to give *p*NPGlc, then another to produce *p*NP. Inspection of the products of a reaction with 5 mM *p*NP-*β*-cellobioside verified that *p*NPGlc was the first product in the initial reaction stage (5–20 min), and no *p*NP nor cellobiose was seen (Figure 7). This indicated that the initial hydrolysis happens between the two glucosyl residues, not at the bond between *p*NP and cellobiose, similar to rice BGlu1 (Os3BGlu7) and GH5BG [30], [31]. However, rather than seeing glucose released from the *p*NPGlc, the other initial product migrated slightly slower than glucose and had absorbance under UV light, suggesting it contains a *p*NP group. Thus, this product was tentatively identified as a *p*NP-glucotrioside transglycosylation product, which would explain the inhibition at higher *p*NP-*β*-cellobioside concentrations, since both the *p*NP-Glc product and *p*NP-*β*-cellobioside could act as acceptors for glucosyl moieties. In fact, when the concentration of the *p*NP-*β*-cellobioside was increased, the release of glucose and *p*NP by rOs1BGlu4 decreased, indicating a significant substrate inhibition, which is consistent with a competing transglycosylation reaction.

### Transglycosylation activity of rOs1BGlu4

**Figure 8:**
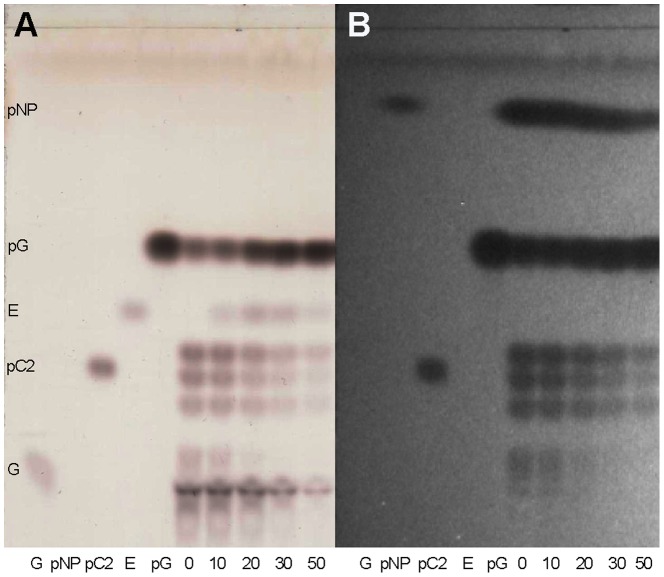
Transglycosylation activity of rOs1BGlu4 with *p*NPGlc and ethanol. The silica gel TLC shows the standards: G, glucose, pG, *p*NPGlc, pC2, *p*NP-*β*-cellobioside, E, ethyl-*β*-D-glucoside; and the reactions: 0 is a reaction with enzyme and 10 mM *p*NPGlc as both donor and acceptor; and 10, 20, 30, 50 are reactions including 10%, 20%, 30% and 50% (v/v) ethanol in addition to 10 mM *p*NPGlc and enzyme. A: TLC plate visualized by the sulfuric acid carbohydrate staining method. B: TLC plate visualized by fluorescence.

The substrate inhibition by *p*NP-*β*-cellobioside and production of an apparent transglycosylation product at 5 mM substrate concentration in Figure 7 suggested that Os1BGlu4 can catalyze transglycosylation at moderate substrate concentrations. It is known that many glycoside hydrolases have transglycosylation activity as well as hydrolytic activity [32]–[34]. When rOs1BGlu4 was incubated with 10 mM *p*NPGlc, several fluorescence-absorbing spots with mobilities slower than *p*NPGlc, characteristic of *p*NP-oligosaccharides such as the *p*NP-*β*-cellobioside standard, were observed on the TLC (Figure 8, lane 0). In fact, one of these products coeluted with *p*NP-*β*-cellobioside from a regular phase carbohydrate column in HPLC (data not shown). One extra product with mobility just below *p*NPGlc was seen on the TLC when ethanol was added as an acceptor substrate (Figure 8 lanes 10, 20, 30, and 50). This new product did not adsorb the UV-induced fluorescence of the TLC plate and migrated at a position identical to ethyl *β*-d-glucoside. Increasing the ethanol concentration from 10% to 20% resulted in more product, but raising it to 30% did not further increase the intensity of this product, likely due to loss of enzyme activity, which was almost completely lost in 50% ethanol. These new products verify the transglycosylation activity of rOs1BGlu4.

**Figure 9:**
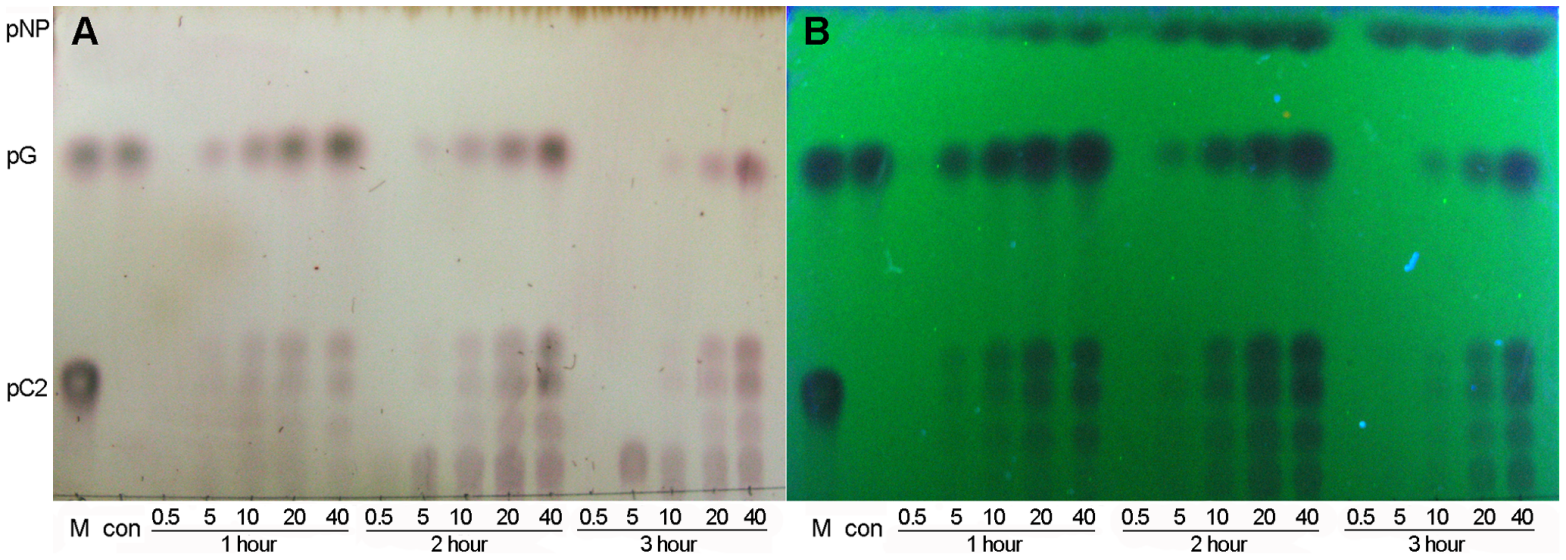
The effects of the different incubation times and substrate concentrations on the transglycosylation activity of Os1BGlu4. TLC analysis of products are shown. The standards are marked as M: marker, including *p*NPGlc (pG), *p*NP-*β*-cellobioside (pC2). *p*NP marks the position of *p*-nitrophenol. For the reactions, con is a control reaction without rOs1BGlu4, and 0.5–40, stand for reactions including 0.5 mM *p*NPGlc, 5 mM *p*NPGlc, 10 mM *p*NPGlc, 20 mM *p*NPGlc, and 40 mM *p*NPGlc, respectively, while 1, 2 and 3 hours are the incubation times. A: TLC plate visualized by the carbohydrate staining method. B: TLC plate visualized by UV light.

The effects of different incubation times and substrate concentrations on the transglycosylation were investigated. At low *p*NPGlc concentration (0.5 mM), at all incubation times, no visible tranglycosylation product can be seen on the TLC (Figure 9). For 5 mM *p*NPGlc, some transglycosylation products can be seen after 1 h incubation, and the products become less after 2 h incubation, and invisible after 3 h incubation. In contrast to the transglycosylation products, the glucose and the *p*NP increased gradually. In reactions with 10 mM, 20 mM and 40 mM *p*NPGlc concentrations, the transglycosylation products increased up to 2 h, then decreased as the *p*NPGlc donor substrate decreased and the equilibrium shifted in favor of their hydrolysis. From the TLC profile, the amount of transglycosylation product was closely related to the *p*NPGlc concentration. At higher concentrations of *p*NPGlc, more transglycosylation product can be observed (Figure 9B).

### Os1BGlu4 subcellular localization analysis

**Figure 10:**
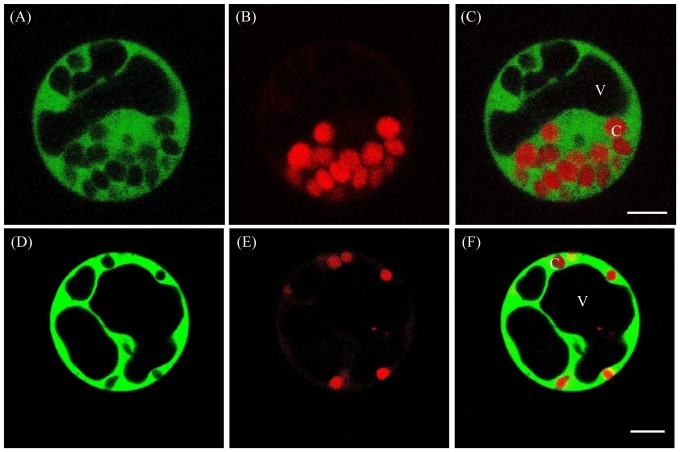
The subcellular localization of Os1BGlu4-GFP and GFP-Os1BGlu4. Subcellular localization of Os1BGlu4-GFP (A–C) and GFP- Os1BGlu4 (D-F) fusion proteins in maize protoplasts. Fluorescent GFP signals (A, D), chlorophyll autofluorescence (B, E) and merged images (C, F) are shown. C, chloroplast; V, vacuole. The bar in the merged images represents 5 µm.

Because the hydrolysis of cell wall-derived oligosaccharides is unexpected for a cytoplasmic enzyme, the localization of Os1BGlu4 was verified experimentally. To determine the subcellular localization of Os1BGLu4, we generated the GFP fusion constructs *GFP-Os1BGlu4* and *Os1BGlu4-GFP* under the control of *CaMV35S* promoter. Results of the subcellular localization experiments showed that the signals of both *GFP-Os1BGlu4* and *Os1BGlu4-GFP* fusion proteins were mainly present in the cytosol of maize protoplasts (Figure 10).

We also introduced the same fusions *GFP-Os1BGlu4* and *Os1BGlu4-GFP* in tobacco epidermal cells. As a result, we found strong signals in both the cytosol and the nucleus as the propidium iodide stained nucleus was overlapped with the GFP signal of the fusion proteins (Figure S1). Western blotting with anti-GFP antibodies showed that the fusion protein was still intact in these cells. These data suggest that Os1BGlu4 is mainly localized in the cytosol in monocot maize cells, while it likely retains a dual targeting ability to both the cytosol and the nucleus in the heterologous dicot tobacco cells.

### Possible biological functions of rice *Os*1*bglu*4 β-glucosidase

The EST profile showed that *Os*1*bglu*4 mRNA is expressed in shoot, root, stem, leaf and callus of rice, which means the Os1BGlu4 may be involved in many physiological processes. The capability of Os1BGlu4 to hydrolyze cello-oligosaccharides, laminaribiose and laminaritriose indicated that the Os1BGlu4 may be involved in cell wall related processes. However, the cytoplasmic localization of Os1BGlu4 makes a direct role in the cell wall unlikely. It is possible that Os1BGlu4 can hydrolyze oligosaccharides which are taken up into the cell by a membrane transporter after their release from cell wall. The release of the glucose inside the cell may be an efficient means to supply the cell with energy.

**Figure 11:**
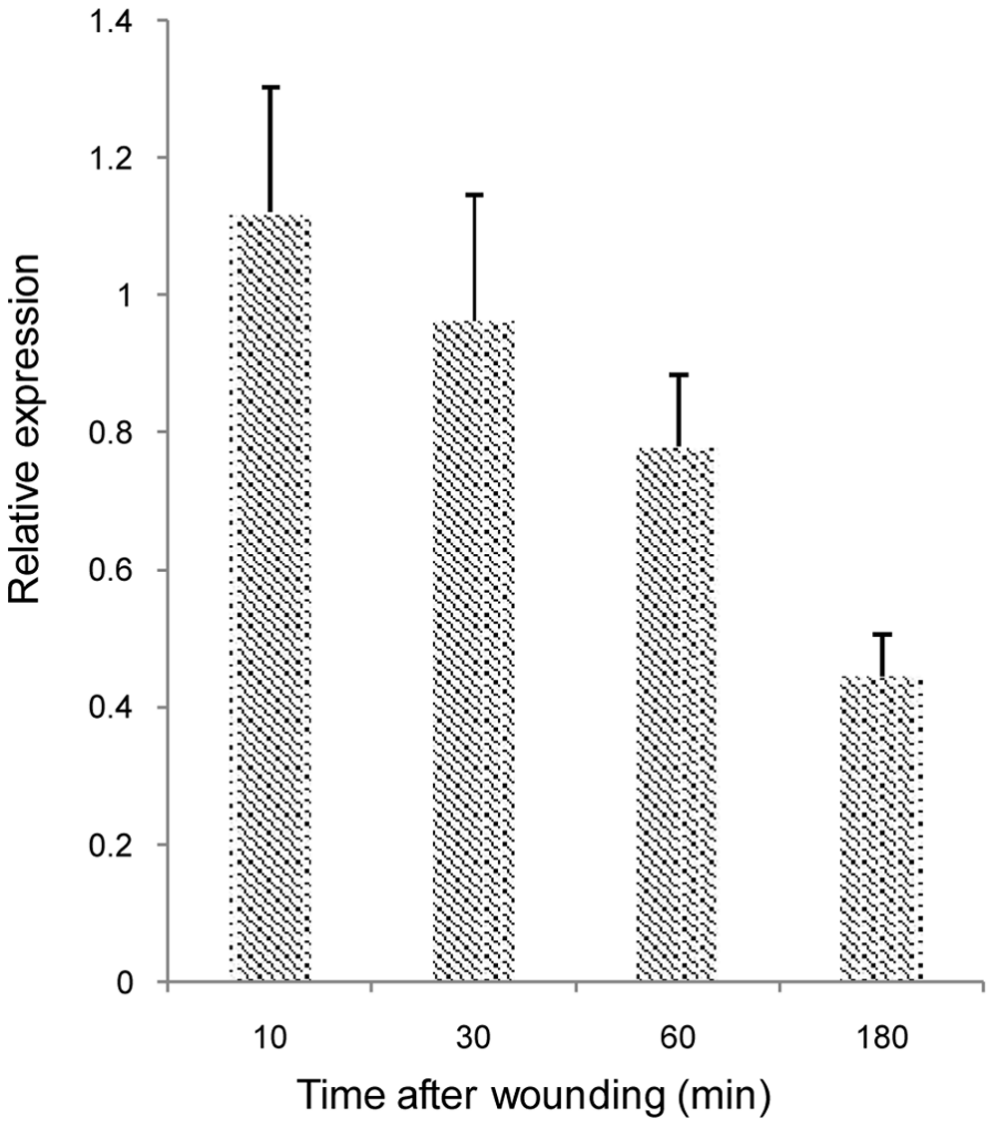
The relative expression of *Os1bglu4* under wounding stress. The stressed *Os1bglu4* expression was determined by quantitative real-time RT-PCR relative to untreated rice with actin as a control gene at various numbers of minutes (min) after wounding of 10 day old rice seedling shoots. The data are given as mean + SE.

Alternatively, the Os1BGlu4 could be released in case of injuries lysing the cells to release the enzyme and contribute to cell wall remodeling and defense at the site of injury. The capability of Os1BGlu4 to hydrolyze salicin and esculin suggested that it may be involved in plant pathogen and herbivore resistance, since the product of hydrolysis of salicin is similar to salicylic acid, which is recognized as an endogenous regulatory signal in plants mediating plant defense against pathogens [35], [36]. The *Os1bglu4* gene EST expression profile indicated it is expressed after blast fungus infection, which could potentially release it from its cytoplasmic location [4]. We tested the hypothesis that Os1BGlu4 could act in response to wounding by quantitative RT-PCR, but the expression seemed to be down-regulated in the first several hours after wounding (Figure 11). Therefore, a role in wounding response seems unlikely.

Aside from its biological function, Os1BGlu4 shows potential for synthesis of glycosides and oligosaccharides. The transglycosylation activity of rOs1BGlu4 suggests that it might be useful for synthesis of *p*NP-oligosaccharides and alkyl glucosides, although further work is necessary to determine the product specificity in transglycosylation.

## Supporting Information

Figure S1 **Subcellular localization of Os1BGlu4-GFP (A-D) and GFP-Os1BGlu4 (E-H) fusion proteins in tobacco epidermal cells and western blot analysis of the Os1BGlu4-GFP and GFP-Os1BGlu4 fusion proteins.** Fluorescent GFP signals (A, E), propidium iodide stained nuclei (B, F) chlorophyll autofluorescence (C, G) and merged images (D, H) are shown. (I) Western blot analysis of Os1BGlu4-GFP and GFP-Os1BGlu4 fusion proteins extracted from the leaf cells with an anti-GFP antibody, which shows that the protein is intact and no cleaved GFP is detectable. C, chloroplast; N, nucleus; V, vacuole. The bar in the merged images, 10 µm.
(TIF)
