# Supplementary figures and images for: Recombinant Expression and Characterization of the Cytoplasmic Rice β-Glucosidase Os1BGlu4
Source: PLoS One. 2014 May 6;9(5):e96712. doi: 10.1371/journal.pone.0096712 (PMC4011751; doi:10.1371/journal.pone.0096712)

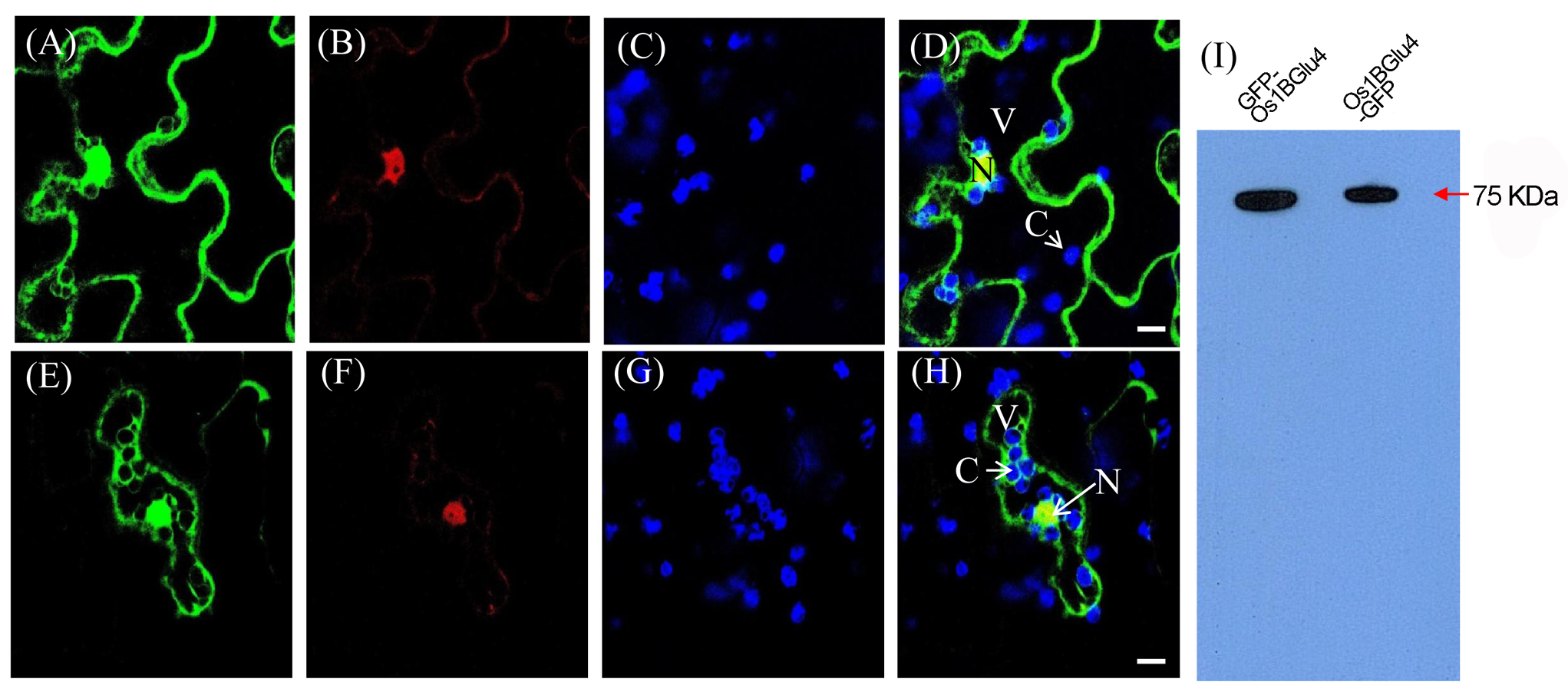

Supplement: Figure S1 — Subcellular localization of Os1BGlu4-GFP (A-D) and GFP-Os1BGlu4 (E-H) fusion proteins in tobacco epidermal cells and western blot analysis of the Os1BGlu4-GFP and GFP-Os1BGlu4 fusion proteins. Fluorescent GFP signals (A, E), propidium iodide stained nuclei (B, F) chlorophyll autofluorescence (C, G) and merged images (D, H) are shown. (I) Western blot analysis of Os1BGlu4-GFP and GFP-Os1BGlu4 fusion proteins extracted from the leaf cells with an anti-GFP antibody, which shows that the protein is intact and no cleaved GFP is detectable. C, chloroplast; N, nucleus; V, vacuole. The bar in the merged images, 10 µm. (TIF) [file pone.0096712.s001.tif]
